# Supplementary material for: Predictors of mortality among inpatients in COVID-19 treatment centers in the city of Butembo, North Kivu, Democratic Republic of Congo
Source: PLOS Glob Public Health. 2024 Jan 24;4(1):e0002020. doi: 10.1371/journal.pgph.0002020 (PMC10807785; doi:10.1371/journal.pgph.0002020)
Supplement: S1 Table — (DOCX) [file pgph.0002020.s001.docx]

S1 Table: Test of proportional hazards assumption

|  | rho | chi2 | df | Prob>chi2 |
| --- | --- | --- | --- | --- |
| Delay between symptom onset and hospital admission | 0.00984 | 0.01 | 1 | 0.9349 |
| Age (years) | -0.18179 | 2.44 | 1 | 0.1183 |
| sex | -0.09883 | 0.76 | 1 | 0.3818 |
| Treatment site | 0.06364 | 0.35 | 1 | 0.5557 |
| History of at least one co-morbidity | -0.08350 | 0.55 | 1 | 0.4566 |
| Stage according to SpO2 | -0.01315 | 0.02 | 1 | 0.9000 |
| m-NEWS | -0.05830 | 0.31 | 1 | 0.5806 |
| Age (≥ 60)#History of at least one co-morbidity | -0.15398 | 1.69 | 1 | 0.1937 |
| Global test |  | 7.32 | 8 | 0.5020 |
