# Supplementary figures and images for: Predictors of mortality among inpatients in COVID-19 treatment centers in the city of Butembo, North Kivu, Democratic Republic of Congo
Source: PLOS Glob Public Health. 2024 Jan 24;4(1):e0002020. doi: 10.1371/journal.pgph.0002020 (PMC10807785; doi:10.1371/journal.pgph.0002020)

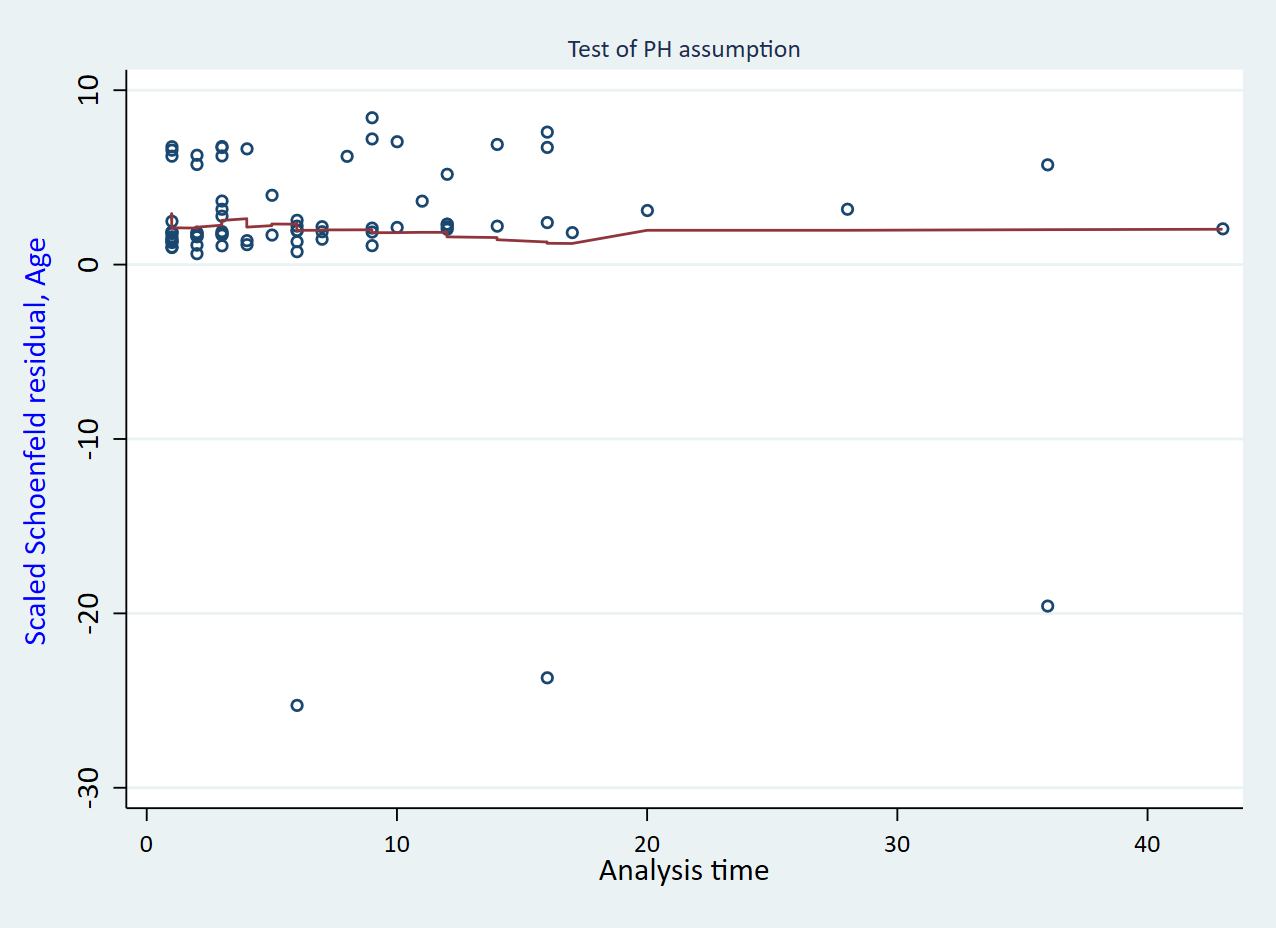

Supplement: S1 Fig — (ZIP) [file pgph.0002020.s002.zip › S1 Fig/Test of PH assumption for Age.tif]

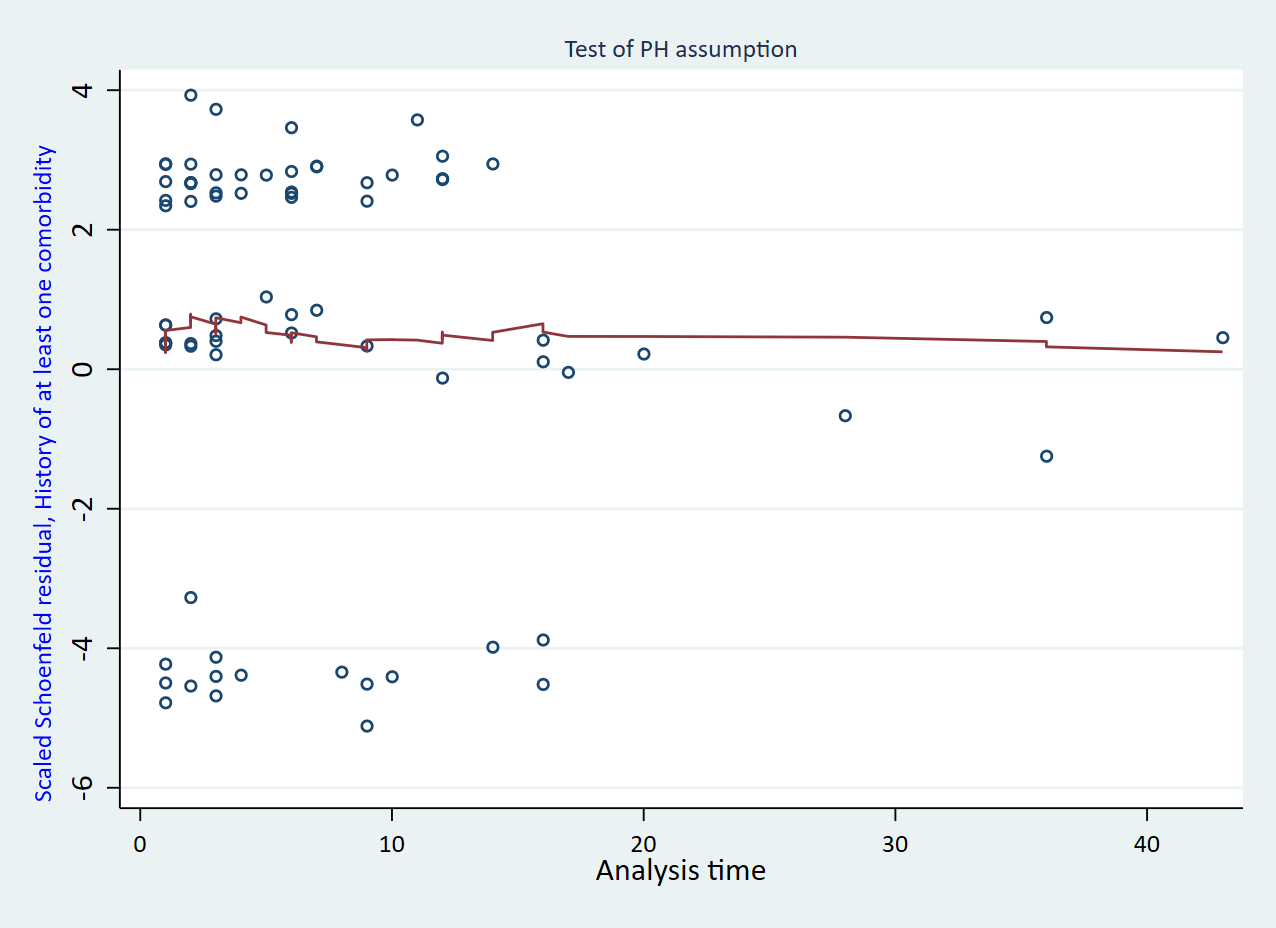

Supplement: S1 Fig — (ZIP) [file pgph.0002020.s002.zip › S1 Fig/Test of PH assumption for Comorbidity.tif]

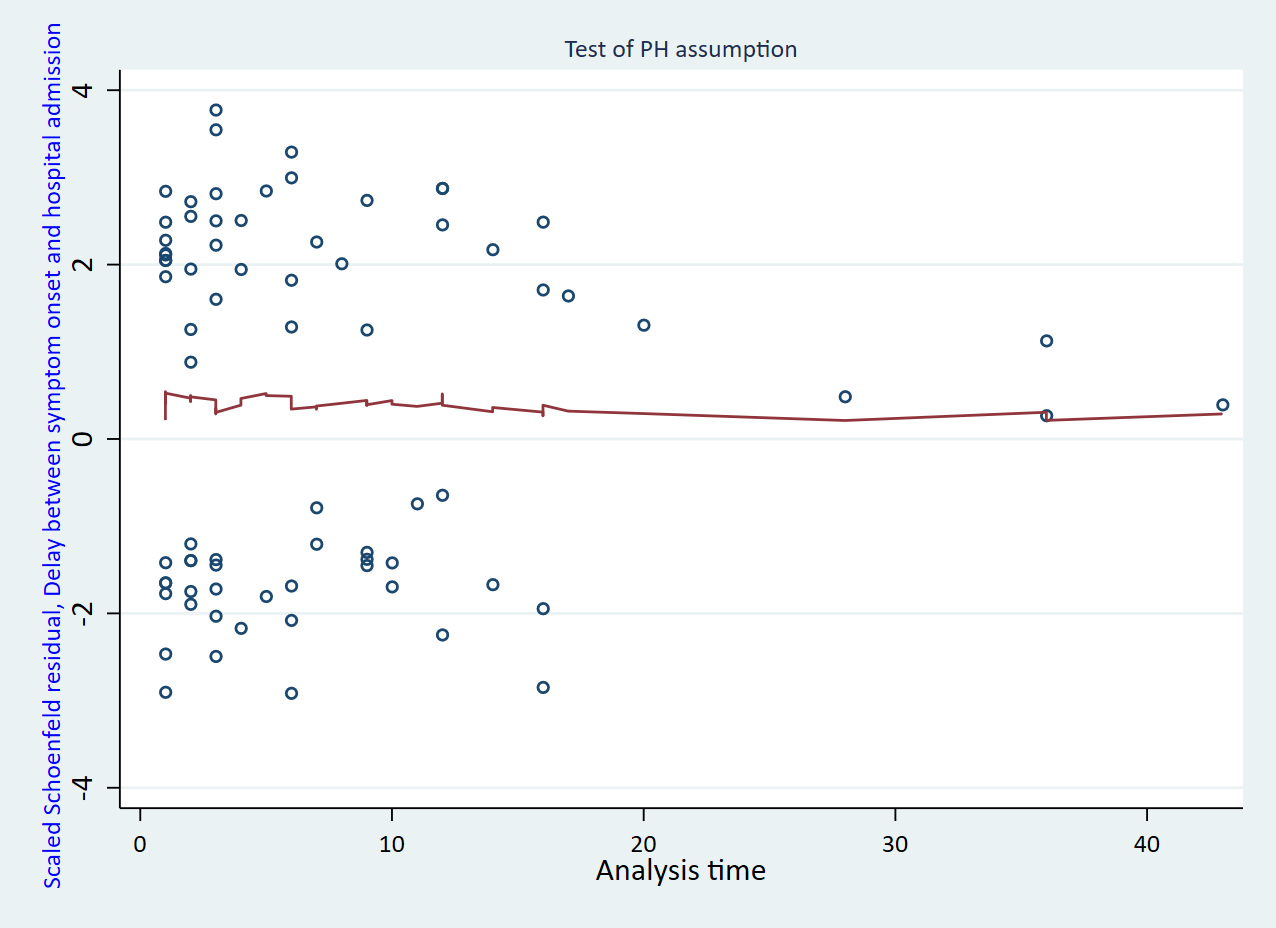

Supplement: S1 Fig — (ZIP) [file pgph.0002020.s002.zip › S1 Fig/Test of PH assumption for Delay between symptom onset and hospital admission.tif]

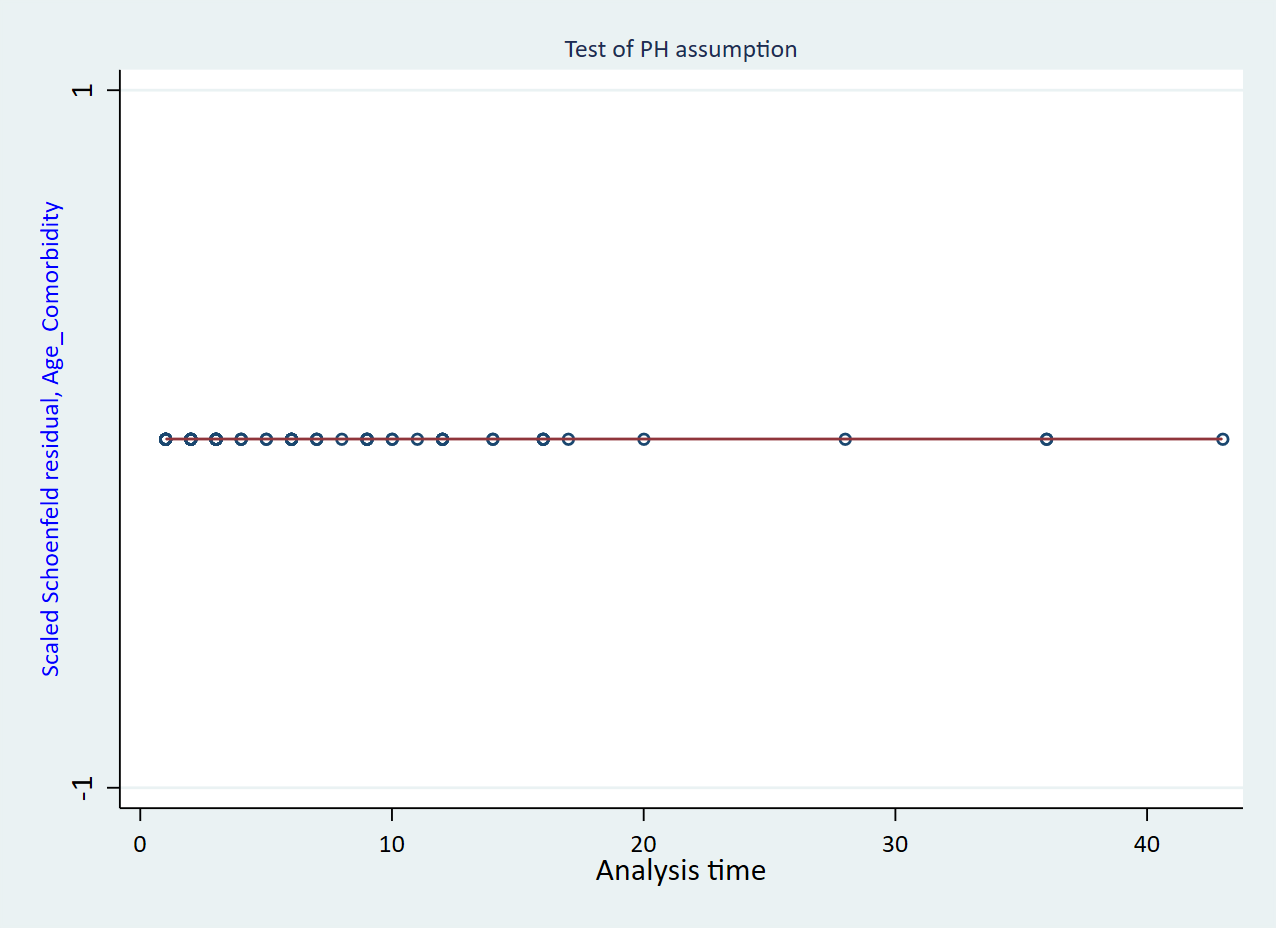

Supplement: S1 Fig — (ZIP) [file pgph.0002020.s002.zip › S1 Fig/Test of PH assumption for interraction Age and comorbidity.tif]

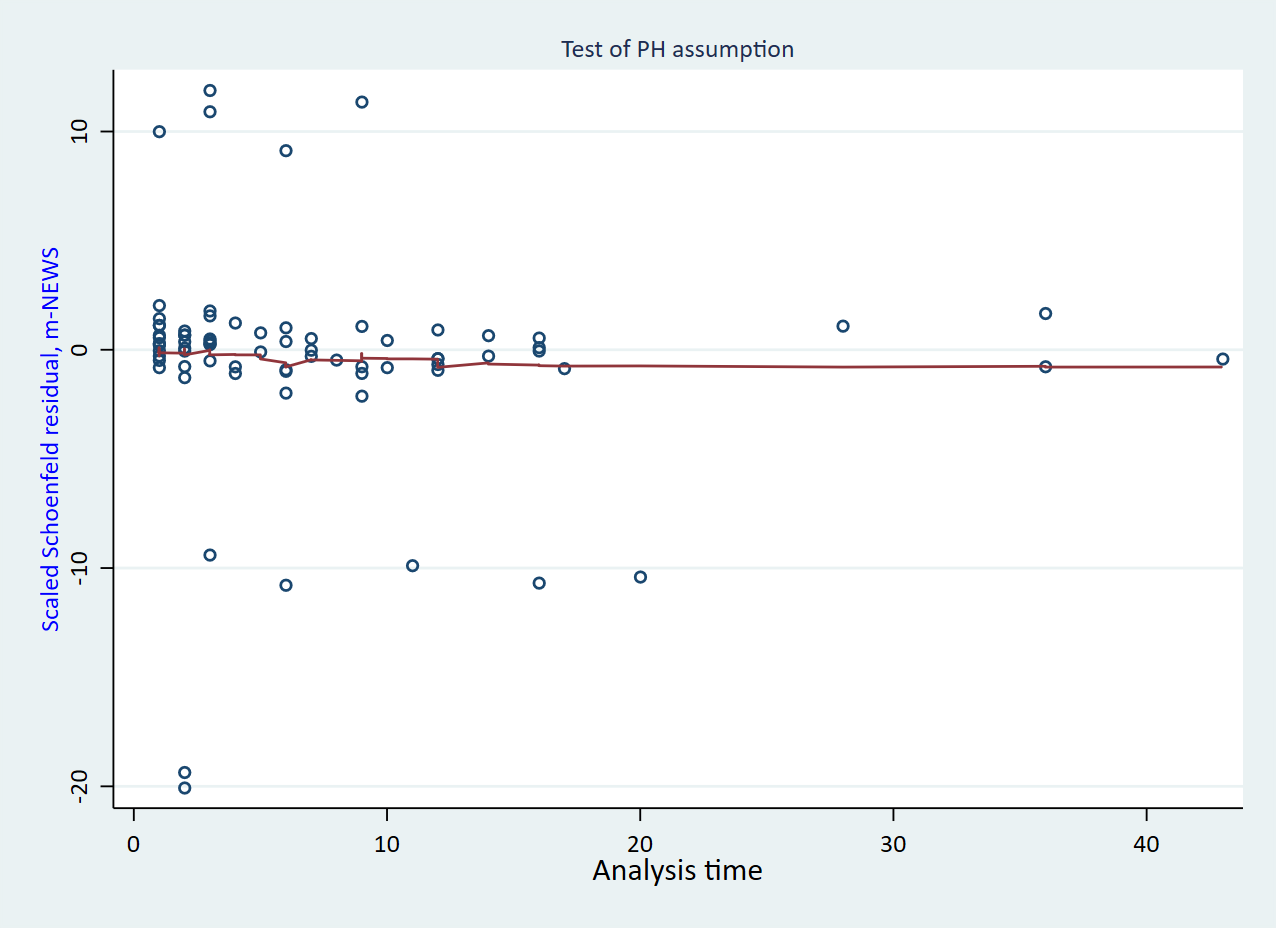

Supplement: S1 Fig — (ZIP) [file pgph.0002020.s002.zip › S1 Fig/Test of PH assumption for m-NEWS.tif]

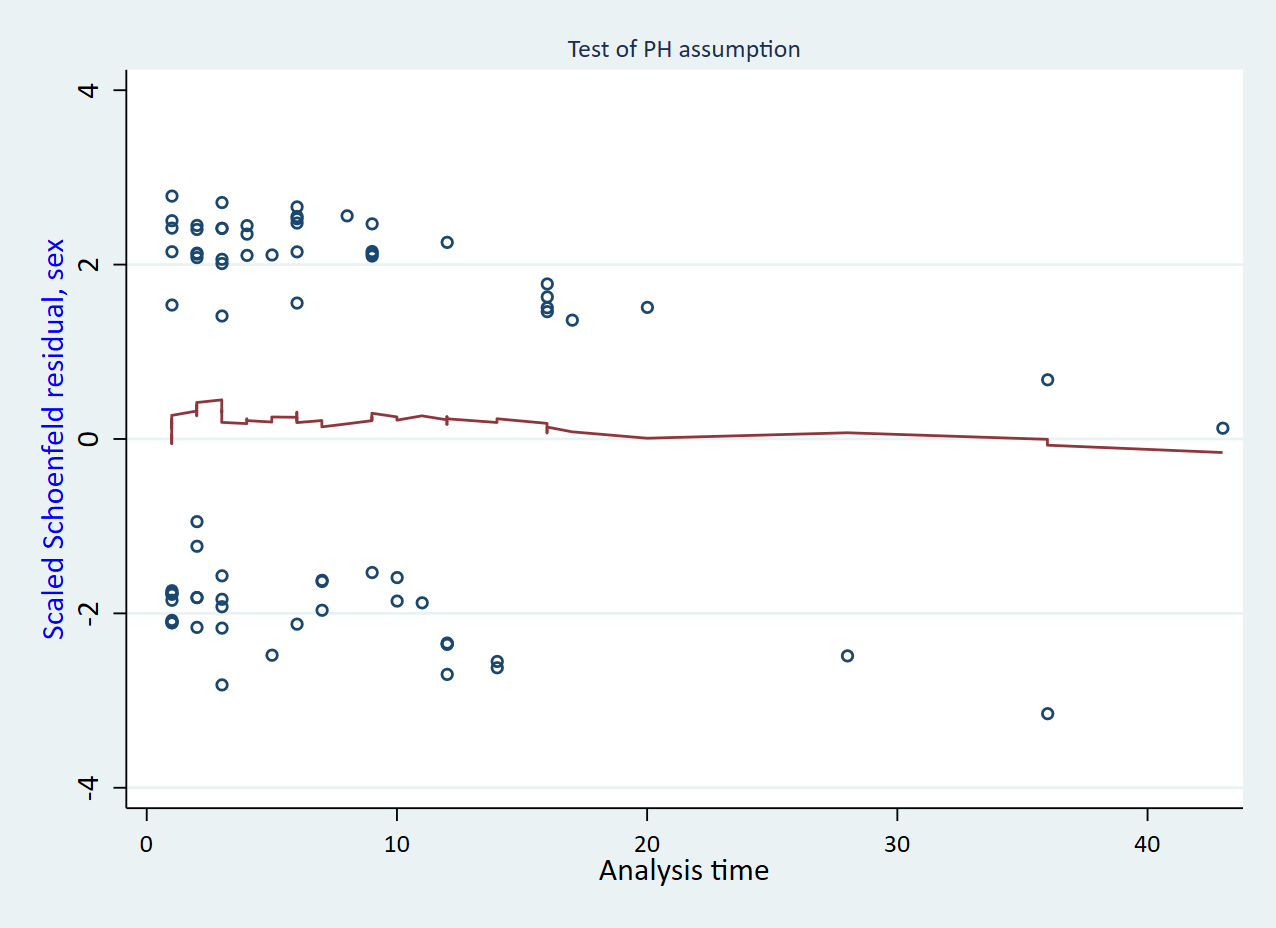

Supplement: S1 Fig — (ZIP) [file pgph.0002020.s002.zip › S1 Fig/Test of PH assumption for Sex.tif]

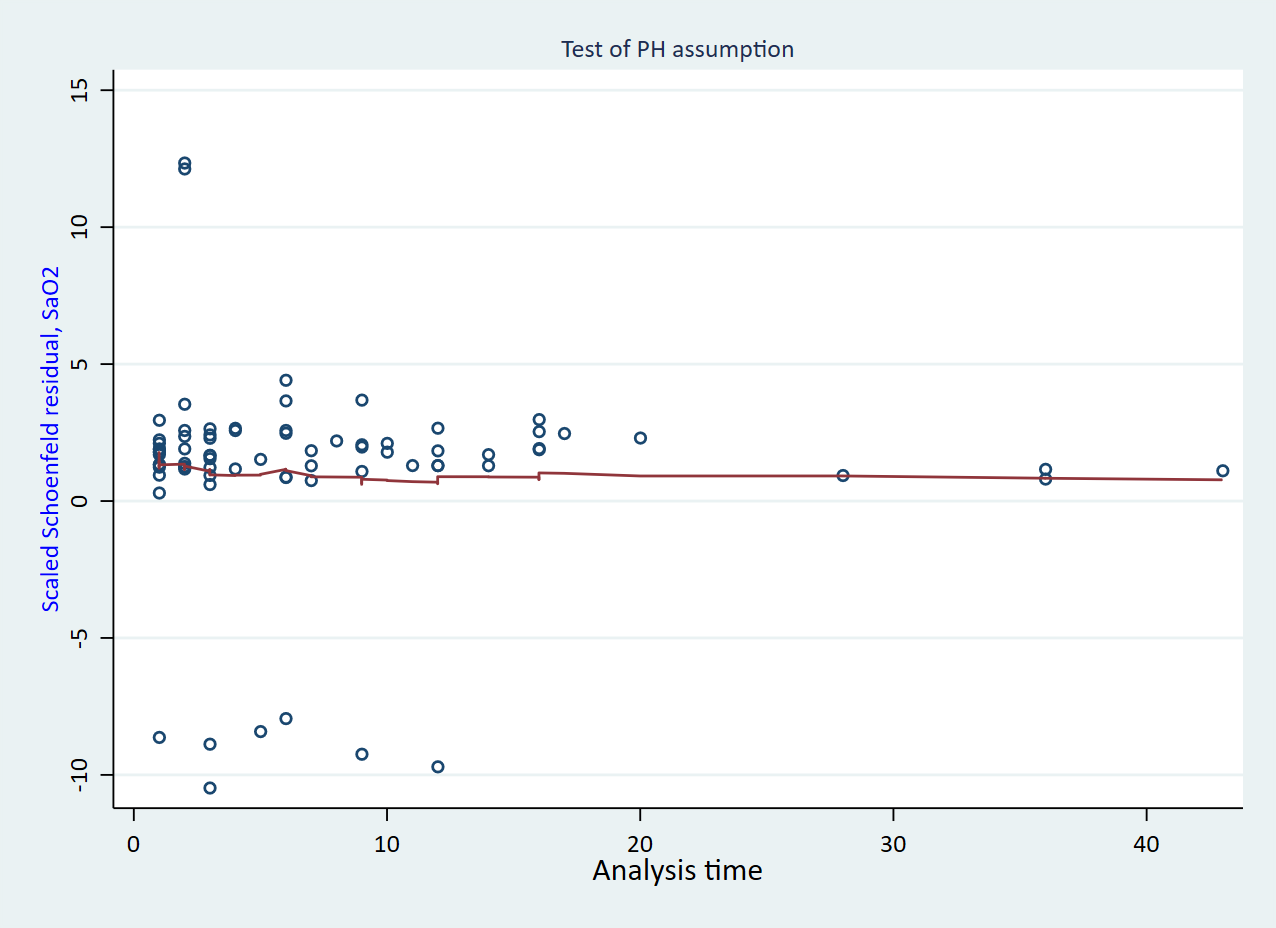

Supplement: S1 Fig — (ZIP) [file pgph.0002020.s002.zip › S1 Fig/Test of PH assumption for Stage according to SpO2.tif]

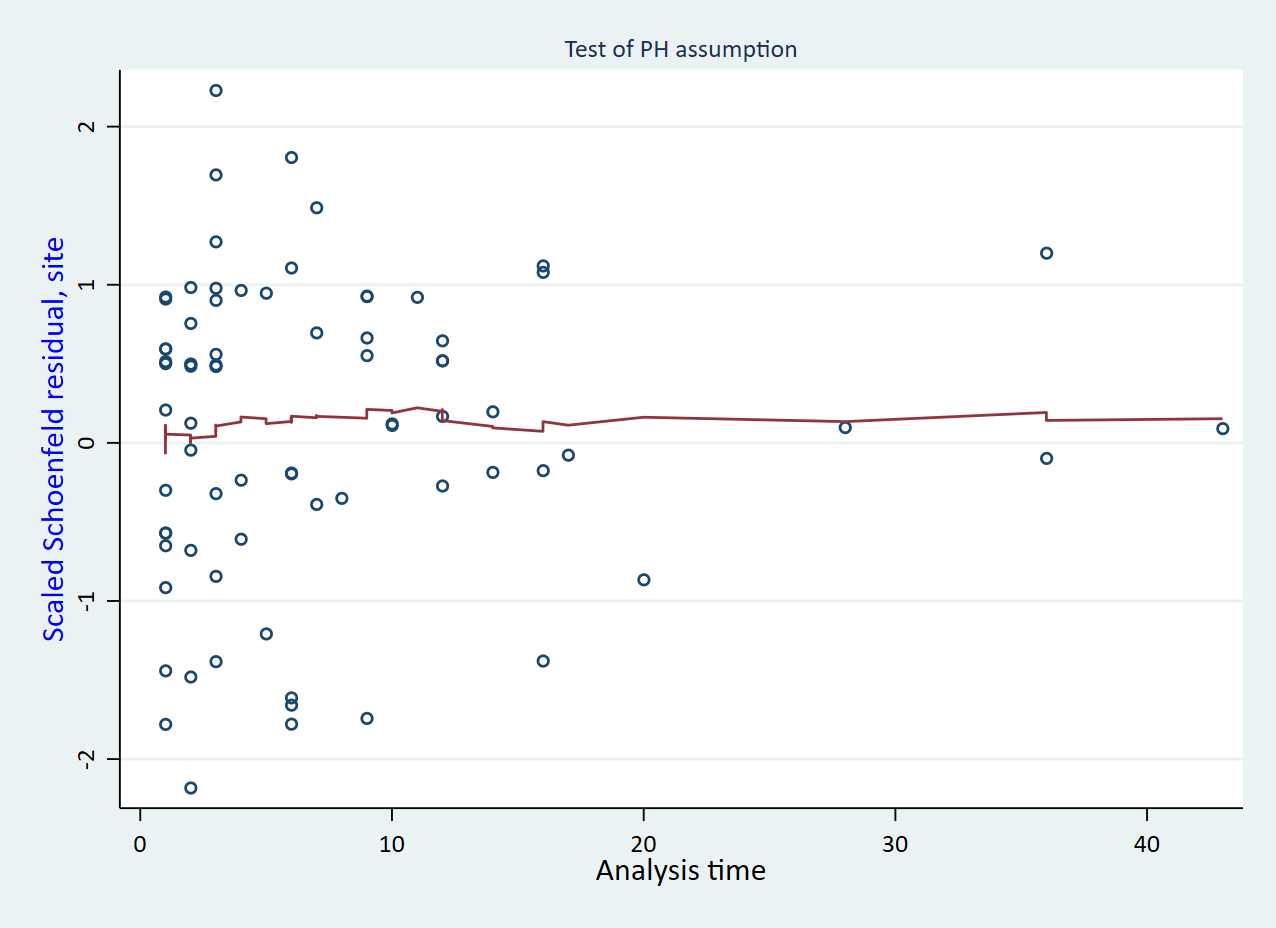

Supplement: S1 Fig — (ZIP) [file pgph.0002020.s002.zip › S1 Fig/Test of PH assumption for Treatment site.tif]

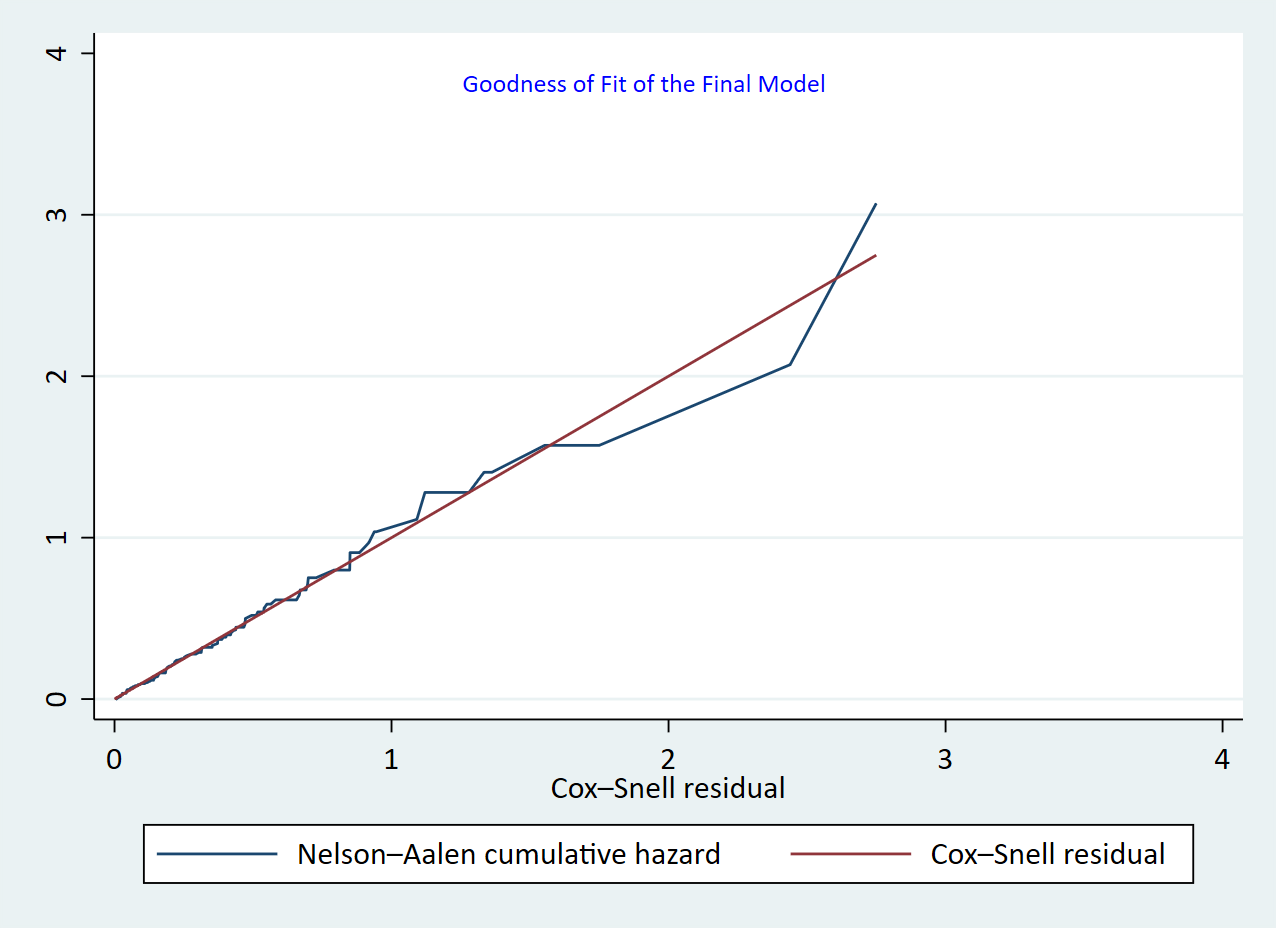

Supplement: S2 Fig — (TIF) [file pgph.0002020.s003.tif]
