# Supplementary material for: Predictors of mortality among inpatients in COVID-19 treatment centers in the city of Butembo, North Kivu, Democratic Republic of Congo
Source: PLOS Glob Public Health. 2024 Jan 24;4(1):e0002020. doi: 10.1371/journal.pgph.0002020 (PMC10807785; doi:10.1371/journal.pgph.0002020)
Supplement: Attachment — Submitted filename: Authors response letter_May13.docx [file pgph.0002020.s004.docx]

RESPONSE LETTER

PGPH-D-23-00180
Predictors of Survival among Inpatients in COVID-19 Treatment Centers in the City of Butembo, North Kivu, Democratic Republic of Congo
PLOS Global Public Health

Dear Editor and Reviewers,

We are immensely grateful for your comments, which help enhance the clarity and contents of our manuscript.

We have addressed your comments point by point. To ease your review process, we have also highlighted the amended text in yellow and pasted it under the respective answers.

With much appreciation for your review and best wishes,

P. Akilimali, on behalf of the co-authors

Journal Requirements:

1. In the online submission form, you indicated that your data will be submitted to a repository upon acceptance.  We strongly recommend all authors deposit their data before acceptance, as the process can be lengthy and hold up publication timelines. Please note that, though access restrictions are acceptable now, your entire data will need to be made freely accessible if your manuscript is accepted for publication. This policy applies to all data except where public deposition would breach compliance with the protocol approved by your research ethics board. If you are unable to adhere to our open data policy, please kindly revise your statement to explain your reasoning and we will seek the editor's input on an exemption. Please be assured that, once you have provided your new statement, the assessment of your exemption will not hold up the peer review process.

**Authors’ response:** Dataset was submitted to dryad

2. Please send a completed 'Competing Interests' statement, including any COIs declared by your co-authors. If you have no competing interests to declare, please state "The authors have declared that no competing interests exist". Otherwise please declare all competing interests beginning with the statement "I have read the journal's policy and the authors of this manuscript have the following competing interests:"

**Authors’ response:** "The authors have declared that there are no competing interests."

3. If you did not receive any funding for this study, please simply state: “The authors received no specific funding for this work.”"

**Authors’ response:** "The authors have not received any specific funding for this work."

Reviewers' comments:

Reviewer's Responses to Questions

**Comments to the Author**

1. Does this manuscript meet PLOS Global Public Health’s [publication criteria](https://journals.plos.org/globalpublichealth/s/criteria-for-publication)? Is the manuscript technically sound, and do the data support the conclusions? The manuscript must describe methodologically and ethically rigorous research with conclusions that are appropriately drawn based on the data presented.

Reviewer #1: Partly

Reviewer #2: Partly

2. Has the statistical analysis been performed appropriately and rigorously?

Reviewer #1: Yes

Reviewer #2: No

3. Have the authors made all data underlying the findings in their manuscript fully available (please refer to the Data Availability Statement at the start of the manuscript PDF file)?

The [PLOS Data policy](https://journals.plos.org/globalpublichealth/s/data-availability) requires authors to make all data underlying the findings described in their manuscript fully available without restriction, with rare exception. The data should be provided as part of the manuscript or its supporting information, or deposited to a public repository. For example, in addition to summary statistics, the data points behind means, medians and variance measures should be available. If there are restrictions on publicly sharing data—e.g. participant privacy or use of data from a third party—those must be specified.

Reviewer #1: No

Reviewer #2: Yes

4. Is the manuscript presented in an intelligible fashion and written in standard English?

PLOS Global Public Health does not copyedit accepted manuscripts, so the language in submitted articles must be clear, correct, and unambiguous. Any typographical or grammatical errors should be corrected at revision, so please note any specific errors here.

Reviewer #1: Yes

Reviewer #2: No

5. Review Comments to the Author

Please use the space provided to explain your answers to the questions above. You may also include additional comments for the author, including concerns about dual publication, research ethics, or publication ethics. (Please upload your review as an attachment if it exceeds 20,000 characters)

**Reviewer #1: Manuscript Summary**

This manuscript identifies predictors of COVID-19 mortality among hospitalized patients in the Democratic Republic of Congo. The authors use cox regression to identify factors that are associated with high mortality rate accounting for time-dependence observed outcome (death). They show that mortality risk is likely influenced by older age, presence of comorbidity, self-defined duration of illness before hospitalization, and ambient air SpO2. Risk factors for severe COVID-19 as well as mortality from COVID-19 have widely been discussed and this study does not add new evidence to the discussion. However, authors claim their study was undertaken at the time when other studies had not yet published their work. On the positive outlook, there are limited studies in Africa describing COVID-19 experiences in hospital settings and this study may be relevant for making a case for experiences in resource-constrained and a humanitarian setting. There are major revisions that need to be made for scientific soundness. Some statistical analysis consideration have been suggested. And lastly, ethics statement is not very clear.

Since line numbering was not put in the article I will describe where suggested changes may go;

**Authors’ response: many thanks for your detailed review and proposed changes in view of improving our manuscript. Much appreciated.**

Title
1.    The authors should modify the title to “Predictors of mortality among ….” And not “Predictors of survival among ….” because that is what they present in the manuscript. The authors should also make changes on every occasion where this occurs in main text.

**Authors’ response: we amended the text, which now reads as follows:**

Predictors of Mortality in patients hospitalized in COVID-19 Treatment Centers in the City of Butembo, North Kivu, Democratic Republic of Congo

Abstract
2.    In the abstract, SpO2 should be written in full before abbreviation

**Authors’ response: we amended the text, which now reads as follows ():**

**Results** – Among the 303 participants (mean age of 53 years), the fatality rate was 33.8 deaths per 1000 patient-days. Four predictors were independently associated with inpatient death: age category (≥ 60 years) (aHR: 4.057; 95% CI:1.61 - 10.27), presence of at least one comorbidity (aHR: 2.757; 95% CI: 1.59 - 4.75); duration of illness of > 5 days before hospitalization (aHR:1.827, 95% CI:1.13 - 2.94) and peripheral capillary oxygen saturation (SpO2) < 90% (HRa=13.16, 95% CI:2.08 - 83.25).

3.    I think the appropriate epidemiologic study design is retrospective cohort study or prospective cohort study as was not clear but not analytic cohort study.

**Authors’ response: we amended the text, which now reads as follows (lines 24-25):**

**Methods** – A retrospective cohort study was conducted in 6 Covid-19 treatment centers in the city of Butembo from 1 January to 31 December 2021.

4.    All considered predictors should be listed in the Methods section so can have assess what went into the models

**Authors’ response: we amended the text, which now reads as follows (lines XX onward):**

**Methods** – The following variables were considered for inclusion to the Cox regression model:  Age, Sex, Disease length, Treatment site, History of at least one co-morbidity, Body mass index, Stage according to SpO2 and the NEWS-modified score.

5.    The wide confidence interval for SpO2 suggests few data points considered, which may likely cause imbalance in model estimation, and authors could ensure to refit models without this variable to assess the impact of this variable

**Authors’ response: we amended the text, which now reads as follows (lines XX onward):**

**Copier les modifications du manuscrit (si indiqué)**

**We had inconsistent data in one category with** a small number of events (**only 2 events (mild group)) resulting to high** standard deviation (standard error **which can explain this wide confidence intervals. This is definitively retained as one of the weekness. We redefine** SpO2 to two categories.

Introduction
6.    Paragraph 5: The word reported must precede these statements as undocumented patients are likely more than the presented numbers.
a.    with a wide variation in the **reported** proportion of patients with severe disease and death
b.    **Reported** morbidity accounts for 6% of cases in the Eastern Mediterranean region.

**Authors’ response: we amended the text, which now reads as follows (lines XX onward):**

The signs and symptoms of COVID-19 vary considerably, with clinical features ranging from asymptomatic presentation to fatal respiratory distress and multiorgan failure [10, 11]. COVID-19 does not exert an equal impact on the different affected regions in the world, with a wide variation in the reported proportion of patients with severe disease and death [12,13]. Reported morbidity accounts for 6% of cases in the Eastern Mediterranean region, 30% in Europe, 39% in the American region, 19% in South Asia, 3% in the Pacific and 4% in Africa [4, 14]. The consequences are palpable worldwide at all levels with significant economic, social, health and financial costs [15, 90].

7.    When was Covid 19 vaccine introduced in this setting? And if at this time it was already introduced, were some patients vaccinated and others not prior to hospitalization? May be good to discuss COVID-19 vaccination situation in this setting.

**Authors’ response: we amended the text, which now reads as follows (lines XX onward):**

As we had collected data on the patient files, information on vaccination against Covid-19 was not included in any patient record. Nevertheless, it is unlikely that there were vaccinated patients among our statistical units, because until the end of December 2021, the city of Butembo had less than 50 vaccinated patients.

Methods
8.    Paragraph 1: Could authors discuss the representation of these hospitals with respect to quality standard of care compared to other hospitals in the country.

We can only discuss regarding Butembo context. These are the best hospitals (organization of care, infrastructure, equipment, personnel, etc.) in the city of Butembo. They are the only sites where the Covid-19 treatment centers operated in the city of Butembo!

9.    Paragraph 3: Could authors discuss what proportion of records were excluded. Did this introduce selection bias? and potentially affecting the validity of results?

The patients whose records were excluded represented less than 5% (14 records were excluded) and therefore could not constitute a selection bias.

10.    Paragraph 3: Why 5 days categorization? My understanding is that authors are introducing an exposure of interest variable based on patient self-reported time when symptoms may have commenced before being hospitalized. Then, why did the authors choose to split this duration by 5 days threshold? Why is this duration important? There is need for some justification of choices made.

**Authors’ response: we amended the text, which now reads as follows (lines XX onward):**

They were divided into two groups: 188 patients hospitalized for ≤ 5 days and 115 patients hospitalized for > 5 days. In diseases, the duration of symptoms before hospitalization has an impact on the evolution. In fact, delayed onset of supportive care affects clinical outcomes and host immune-inflammatory and thrombotic responses. And for Cummings MJ et al., had found the average duration of Covid-19 illness in patients before hospitalization of 5.01 days. [44, 58]. This categorization was made also in the previous paper: Dananché C, Elias C, Hénaff L, Amour S, Kuczewski E, Gustin M-P, et al. (2022) Baseline clinical features of COVID-19 patients, delay of hospital admission and clinical outcome: A complex relationship. PLoS ONE 17(1): e0261428. https://doi.org/10.1371/journal.pone.0261428

11.    Data collection: Could authors also justify thresholds used to transform continuous variables into categorical variables

**Authors’ response: we amended the text, which now reads as follows (lines XX onward):**

he transformation of continuous variables into categorical variables was made by referring to previous studies and to the standards of the health information system in the DRC.( reference [21, 22, 23, 24, 25, 26, 27, 28, 29, 30,31, 32]

12.    Statistical analysis: All tests were two-tailed with 95% confidence intervals and considered **statistically** significant when p-value < 0.05.

**Authors’ response: we amended the text accordingly.**

Ethical considerations
13.    The first and last sentences in the first paragraph are irrelevant. Also, the last paragraph is irrelevant. Additionally, I think this section should clearly state if patients consented to participate in the study, and which ethics committee approved the study and the approval number, which in my opinion is not very clear.

**Authors’ response: we amended the text accordingly.**

Results
14.    All presented results do not have referenced table or figure where the readers can refer for further details. Could authors cite relevant table or figure for each paragraph.

**Authors’ response: we amended the text as suggested.**

15.    Paragraph 2: “Regarding comorbidity, 45.9% had at least one and 25.2% (35/139) had at least two” could authors consider rephrasing this sentence as “Of 303 patients with comorbidities, 45.9% had at least one of whom 25.2% had at least two comorbidities.” As it is it’s a bit confusing especially when denominators are not clearly shown.

**Authors’ response: we amended the text, which now reads as follows:**

Of 303 patients included, 45.9% had at least one comorbidity of whom 25.2% had at least two comorbidities.
16.    Paragraph 2: Is it possible to quantify the distribution and write the results in text rather than making descriptive sentence. e.g., <=5d mean X SDX or Median X IQR X-X etc. If the same could be done in all places where the distributions are simply described.

**Authors’ response: we amended the text, which now reads as follows (lines XX onward):**

All results are in table and to reduce the number of words, we used this formula.

17.    Last paragraph: Could author include all reference groups for the estimated predictor level rather than leaving readers guessing what the hazard ratios comparisons are with.

**Authors’ response: we amended the text, which now reads as follows (lines XX onward):**

After adjusting for disease duration, age, gender, history of comorbidity, BMI, treatment site, disease stage by SO2 on admission, m-NEWS, four predictors were independently associated with in-hospital death: older patients (≥ 60 years) had a higher risk of death than the reference group(<40 years) (adjusted HR: 4.057; 95% CI:1.61 - 10.27), having at least one comorbidity had a higher risk of death than patients without comorbidity (aHR: 2.757; 95% CI: 1.59 - 4.75); patient admitted after “long delay” between symptom onset and hospital admission duration of illness of > 5 days before hospitalization had a higher risk of death than patients admitted after “short delay” (aHR:1.827, 95% CI:1.13 - 2.94) and Patients with SpO2 < 90% had a higher risk of death than patients with with SpO2 ≥ 95% (HRa=13.16, 95% CI:2.08 - 83.25)

18.    Table 3: Some percentages are not adding to 100%, and all percentage could be kept to 1 decimal place for consistence.

**Authors’ response: we amended the table accordingly.**

19.    Table 4: Not sure why authors have been calling the exposure of interest different names e.g., Disease length, Disease duration before hospitalization, Time from symptom to hospitalization. It would be good to be consistent with exposure variable name. Also, I guess you meant to bold its p value for being statistically significant

**Authors’ response: we amended the table accordingly.** We are **using now delay between symptom onset and hospital admission.**

20.    In all the plots, the y-axis says: “Cumulative incidence of death” but the y-axis seems to suggest it’s probability and not necessarily incidence (as units are not present and it’s restricted between 0 and 1). It should rather be named “Mortality probability” or if the inverse is applied with curves starting from 1 on downward trend, the y-axis should be named “Survival probability”. I would think the latter is frequently used.

**Authors’ response: we amended the plots accordingly.**

21.    It’s also relevant to include confidence intervals in the survival curves to have a clearly understanding of uncertainty, and if authors are happy to do so.

**Authors’ response: Good idea but in table 4: we have presented HR with confidence intervals and this should help readers.**

Discussion
22.    Paragraph 1: The first paragraph of the Discussion should also discuss the "So what question" e.g., what’s the implication of these key findings or what does it mean now that these factors are identified?

**Authors’ response: we amended the text, which now reads as follows (lines XX onward):**

**Authors’ response: We have edited the first paragraph by adding the implication.**

23.    Paragraph 2: What does it mean, older age groups are more susceptible to infection e.g., is it to acquire SARS-CoV-2 or it is about invasiveness? Could the authors clarify.

**Authors’ response: we amended the text and made this clarification.**

24.    As mentioned beforehand, COVID-19 vaccines have had substantial impact on mortality from COVID-19 disease. A paragraph should be included to discuss the impact of these vaccines on the results.
25.    Authors should also discuss the implication of undocumented COVID-19 deaths in DRC due to resources-constraints and how this could affect their results in this manuscript.

**Authors’ response: During the study period, data related to vaccination were not yet available in Butembo.** And we discussed this in the discussion section.

**Reviewer #2: Manuscript summary**

The paper aims at identifying the predictors of survival for Covid-19 patients in North Kivu province in the Democratic Republic of Congo. This was an observational study conducted in six study sites where time to death was an outcome of interest. In order to determine factors associated with death they used Cox regression model. The study concludes that Advanced age, history of comorbidity, low level of ambient air SpO2 at admission and long duration of the disease before hospitalization were predictors of survival of patients hospitalized for Covid-19. This is an important study that would support formulation of policy based on local evidence. Generally, the authors attempt to present the paper in a simplistic style. This led to loss of important details. Additionally, there seems to be inconsistency in the arrangement of their articles, making it less succinct. Below are specific comments for each section

**Authors’ response: many thanks for your detailed review and proposed changes in view of improving our manuscript. Much appreciated.**

Abstract

1. The background section of the abstract jumps to the relevance/significance of the study. Can the authors provide some introduction leading to this work rather than jumping straight to relevance/significance?
2. In methods section of the abstract, the authors indicate that the study was done from 1 January to 31 December 2021. It doesn’t indicate when the study finished for the current analyses.

**Authors’ response:** Patients included in this analysis was hospitalized from 1 January to 31 December 2021.

Introduction

3. The introduction provides rich information on Covid-19. However, the text flow seems inconsistent: It would be important if the authors may re-arrange their argument based on the key themes e.g. background information, what has already been done, the existing gap and the significance/rationale of the study. As it stands, it is hard to read through the introduction and know the messages that the authors are intending to deliver
4. The paragraphs capturing same concepts seem to be unnecessarily split. For example, it can be observed that paragraphs 3 and 4 capture the same concept. The authors should consider rearranging the paragraphs so that they succinctly capture the concepts without unnecessary breaks.
5. In the first paragraph can the authors provide references for the facts presented about covid-19
6. In paragraph 3, can the authors consider correcting the inconsistency on how the dates are reported. This correction should also to the other sections within the manuscript.

**Authors’ response: we have edited accordingly.**

Methods

7. In paragraph 3 of the methods section, can the authors provide the rationale for dichotomizing length of stay in the hospital using a cut-off of 5 days.

**Authors’ response: we amended the text, which now reads as follows (lines XX onward):**

They were divided into two groups: 188 patients hospitalized for ≤ 5 days and 115 patients hospitalized for > 5 days. In diseases, the duration of symptoms before hospitalization has an impact on the evolution. In fact, delayed onset of supportive care affects clinical outcomes and host immune-inflammatory and thrombotic responses. And for Cummings MJ et al., had found the average duration of Covid-19 illness in patients before hospitalization of 5.01 days. [44, 58]. This categorization was made also in the previous paper: Dananché C, Elias C, Hénaff L, Amour S, Kuczewski E, Gustin M-P, et al. (2022) Baseline clinical features of COVID-19 patients, delay of hospital admission and clinical outcome: A complex relationship. PLoS ONE 17(1): e0261428. https://doi.org/10.1371/journal.pone.0261428

8. The data collection section seems too brief. Can the authors consider adding detailed information e.g. Who collected the data? How did you ensure data quality considering that you used excel that doesn`t provide trails for any changes? How was the validation and verification done? What version of Stata was used for the analysis? How was the transformation done for each respective variable?

**Authors’ response: we amended the text and provide missing information.**

9. The statistical analysis seems too brief. Can the authors consider adding more details e.g. What descriptive statistics where computed and how they were reported, the definition of censoring or loss to follow-up, variable selection procedure/criteria, model selection and diagnostic tests etc.

**Authors’ response: we amended the text and explain all statistical analysis we made.**

10. Under the ethical considerations is ESP/KINSHASA ethic review committee? If no, which ethic review committee approved the study?
11. Under the ethical considerations, the authors should consider formatting their text so that it is consistent.

## Ethical considerations

**Authors’ response:** ESP/KINSHASA IRB served the review committee **we have edited accordingly.**

12. Results section
A. In the results section, can the authors consider reporting the total person time, indicate with the text which variables were adjusted for, consider reporting age median(IQR): it can be observed here that the age is skewed.

**Authors’ response: we have edited accordingly.**

B. In the results section, can the symptoms be further collapsed based on body organs e.g. digestive disorders (ie. Gastrointestinal disorders), neuro-system disorders (e.g headache), renal disorders, respiratory system disorders, co-morbidity etc.

**Authors’ response: we have edited accordingly.**

C. In all the tables decimal should be . not ,

**Authors’ response: we have edited accordingly.**

D. The P-Value cannot be reported as 0, 000. If it is 0.000 it should be reported as <0.001

E. The hazard ratios should be reported to not more than 2 decimal places and this should be consistent across the other estimates reported in the manuscript

**Authors’ response: we have edited accordingly.**

F. It doesn’t make sense that there was an age-group of patients less than 40 years: this less to sparse estimates. Please revisit this and consider collapsing the age categories further

**Authors’ response:** We categorised in three group to analyze also responses regarding young patient(less than 40 years old)

13. Discussion
The authors provide a rich discussion.
A. However, it would be interesting if the authors can highlight what they think are their study observed different findings from the other similar studies [as indicated in their text].

**Authors’ response:** Overall, these predictors have been previously reported in studies conducted in China, Europe, the USA and other African countries with the addition of another new predictor introduced in the present study: the delay between symptom onset and hospital admission.

B. Are there any potentially important potential confounders, not collected in this study that could further be interest in future studies

C. Did you consider how could selection bias could potentially affect the current findings? It seems not fully discussed in the current study. For instance, since this is hospital based-surveillance, it means those patients with poor health seeking behavior could not have been part of this study.

**Authors’ response: we amended the text, which now reads as follows (lines XX onward):**

**Authors’ response:** Data on vaccination that might influence outcome were not available, but also patients with poor care-seeking behavior would be excluded from this study.
