# Supplementary material for: Predictors of mortality among inpatients in COVID-19 treatment centers in the city of Butembo, North Kivu, Democratic Republic of Congo
Source: PLOS Glob Public Health. 2024 Jan 24;4(1):e0002020. doi: 10.1371/journal.pgph.0002020 (PMC10807785; doi:10.1371/journal.pgph.0002020)
Supplement: Attachment — Submitted filename: PGPH-D-23-00180_R1 Review Deus Thindwa.docx [file pgph.0002020.s005.docx]

Summary

The authors have positively responded to substantial number of queries and could be considered for publication. However, I think some minor revisions/additions need to address outstanding issues, per editor’s decision, that may have been overlooked.

1. The wide confidence interval for SpO2: Authors’ response does not seem to resolve the issue. The intervals range between 2.08-83.25, clearly indicating instability. Authors would rather make two categories by combining mild and moderate levels to have at least 10 deaths in each cell or model the variable as continuous than categorical.
2. Ethical considerations statement does not still state the ethics committee that authorized the study. My understanding is that its not enough to have permission from health authorities to do a study without an ethics committee unless health authorities represent the ethics committee, in which case it must be stated, including the approval number.
3. The first paragraph of the Discussion should also discuss the "So what question" e.g., what’s the implication of these key findings or what does it mean now that these factors are identified? Authors say, “Knowledge of these factors is an important advocacy tool for covid patients in humanitarian and resource-limited settings.” Not sure this explicitly states what the implication of the results are, and readers would benefit from understanding how the results would help improve healthcare services.
4. The discussion around COVID-19 vaccines in DRC since April 2021 during the study period has not been fully discussed except for a statement saying the authors did not have records for vaccination. Authors may want to state vaccination coverage by Dec 2021 (during study end) and how this would affect case fatality across time since Apr 2021.
5. The authors have not discussed the implication of undocumented COVID-19 cases and deaths in DRC due to resources-constraints as queried previously. This has substantial impact on study results as not all cases may have ended up to the hospital thereby creating reporting bias of the outcome.
