# Supplementary material for: Predictors of mortality among inpatients in COVID-19 treatment centers in the city of Butembo, North Kivu, Democratic Republic of Congo
Source: PLOS Glob Public Health. 2024 Jan 24;4(1):e0002020. doi: 10.1371/journal.pgph.0002020 (PMC10807785; doi:10.1371/journal.pgph.0002020)
Supplement: Attachment — Submitted filename: Authors response letter_October 22.docx [file pgph.0002020.s006.docx]

RESPONSE LETTER

PGPH-D-23-00180R1
Predictors of Survival among Inpatients in COVID-19 Treatment Centers in the City of Butembo, North Kivu, Democratic Republic of Congo
PLOS Global Public Health

Dear Editor and Reviewers,

We are immensely grateful for your comments, which help enhance the clarity and contents of our manuscript.

We have addressed your comments point by point. To ease your review process, we have also highlighted the amended text in the revised version and pasted it under the respective answers.

With much appreciation for your review and best wishes,

P. Akilimali, on behalf of the co-authors

Reviewer #1: The authors have addressed majority of previous comments, and only minor issues need to be addressed.

Summary

The authors have positively responded to substantial number of queries and could be considered for publication. However, I think some minor revisions/additions need to address outstanding issues, per editor’s decision, that may have been overlooked.

1. The wide confidence interval for SpO2: Authors’ response does not seem to resolve the issue. The intervals range between 2.08-83.25, clearly indicating instability. Authors would rather make two categories by combining mild and moderate levels to have at least 10 deaths in each cell or model the variable as continuous than categorical.

The wide confidence interval for SpO2 is due to the small number of events in the reference group, we have only 2 cases of death in mild severe patients.

2. Ethical considerations statement does not still state the ethics committee that authorized the study. My understanding is that its not enough to have permission from health authorities to do a study without an ethics committee unless health authorities represent the ethics committee, in which case it must be stated, including the approval number.

We have edited the manuscript and added the reference letter:

The protocol for this study had received ethical approval from the School of Health Ethics Committee (reference number: ESP/CE/138/2021).

3. The first paragraph of the Discussion should also discuss the "So what question" e.g., what’s the implication of these key findings or what does it mean now that these factors are identified? Authors say, “Knowledge of these factors is an important advocacy tool for covid patients in humanitarian and resource-limited settings.” Not sure this explicitly states what the implication of the results are, and readers would benefit from understanding how the results would help improve healthcare services.

We have addressed this issue:

The use of NEWS-modified or original NEWS score, the measurement of oxygen saturation and search for co-morbidities must be systematic. These parameters may help to select the most at-risk patients with a poor prognosis and to intensify surveillance. In almost all hospitals in Africa in general, and in the DRC in particular, the measurement of oxygen saturation was not systematically used of times due to the absence of an oximeter.

4. The discussion around COVID-19 vaccines in DRC since April 2021 during the study period has not been fully discussed except for a statement saying the authors did not have records for vaccination. Authors may want to state vaccination coverage by Dec 2021 (during study end) and how this would affect case fatality across time since Apr 2021.

We have addressed this issue:

Regarding COVID-19 vaccines, the roll-out started on April 19^th^, 2021. However, the uptake was slow initially, by the time of data collection, **only about 7%** of the target population (entire population >18) had received at least one dose in the entire country. This low coverage may affect case fatality.

5. The authors have not discussed the implication of undocumented COVID-19 cases and deaths in DRC due to resources-constraints as queried previously. This has substantial impact on study results as not all cases may have ended up to the hospital thereby creating reporting bias of the outcome.

Great! We have reported this statement in the revised manuscript.

Reviewer #3:

Use the word Covid-19 as COVID-19.

We have edited in the current version.

Please report the number of populations in the abstract.

The sample size is set in the abstract: 303 participants.

Please follow reporting guideline such as STOBE

Thanks, and this is done.

It is needed to clarify how the COVID-19 severity was classified. Provide more details about these measures.

Thanks, and this can see in the manuscript:

It allows the classification of Covid-19 patients into three severity categories: low (aggregate 1-4), medium (aggregate 5-6), and high (aggregate 7 or more).

Specify what is the exposure variable.

Delay between symptom onset and hospital admission was the exposure variable. patients were divided into two groups: 188 patients hospitalized for ≤ 5 days(Short delay) and 115 patients hospitalized for> 5 days(long delay).

Provide clear explanation of the time variable of how this was calculated.

The time variable was calculated from the date of enrolment (admission at the hospital) to the end point( death, discharge or end of the study).

Provide the ethical approval number.

The protocol for this study had received ethical approval from the School of Health Ethics Committee (reference number: ESP/CE/138/2021).

Justification of this study is not clearly presented.
Introduction should be more informative at this stage of covid that how this study can add new.
Provide the limitation of the study.

This sis done in the current version.

Reviewer #4:

It is multivariate or multi-variables?

Statistically speaking, multivariate analysis refers to statistical models that have 2 or more dependent or outcome variables,1 and multivariable analysis refers to statistical models in which there are multiple independent or response variables. We have edited and used multivariable analysis.

Please specify if this is which model was used, semi parametric cox proportional hazard model or parametric proportional Cox regression.
The assumption of the semi parametric cox proportional hazard is to be met the proportionality of the hazard.
The assumption of the parametric cox regression is to be met the proportionality of the hazard as well as the distribution of the time variable.
So, specify which model was used in this study.
Do the test for all the assumptions to be met that can be more than one test. For example, for categorical data the test may be not same as for continuous.
Report the graphical presentation of the assumptions. It can be reported as supplementary file so that reader can assess the quality of the assumption as well as the model.
What was done while the proportionality was not met. As I can see in the mortality the KM curve overlap.

We used the semi parametric cox proportional hazard model. The Test of proportional hazards shown that the assumption was not violated as presented in the table and supplementary file

Report how the confounding and interaction among the co-variate was addressed.

The interaction age and SpO2 was not significant and was not included in the model. The interaction between the comorbidity and «the delay between symptom onset and hospital admission» was not significant and was not included in the model. The interaction between age and «the delay between symptom onset and hospital admission» was not significant and was not included in the model. The interaction age and comorbidity was significant and was included in the model. We then compare the model with the interaction to the model without the interaction using the lrtest command. The significant lrtest indicates that we reject the null hypothesis that the two models fit the data equally well and conclude that the bigger model with the interaction fits the data better than the smaller model which did not include the interaction.

Report the model fitness such as dfbeta, devodemce residuals, devicence, or Cox-snell residuals. Please provide everything related to the model fitness in the supplementary file.

We see that the hazard function follows the 45-degree line very closely except for very large values of time. Overall, we would conclude that the final model fits the data very well.
